# A Transformative Wearable Corneal Microneedle Patch for Efficient Therapy of Ocular Injury and Infection

**DOI:** 10.1002/advs.202414548

**Published:** 2025-01-31

**Authors:** Xue Jiang, Shuhua Liu, Jiayi Chen, Jiapeng Lei, Wenjing Meng, Xueyang Wang, Zhigang Chu, Wei Li

**Affiliations:** ^1^ Department of Burns Tongren Hospital of Wuhan University (Wuhan Third Hospital) School of Pharmaceutical Sciences Wuhan University Wuhan 430071 China; ^2^ TaiKang Center for Life and Medical Sciences Wuhan University Wuhan 430071 China; ^3^ Hubei Provincial Key Laboratory of Developmentally Originated Disease Wuhan 430071 China

**Keywords:** contact lens, microneedles, ocular diseases, ocular drug delivery, sustained release

## Abstract

Ocular injury and infection are significant causes of vision impairment and blindness globally. Effective treatment is, however, challenging due to the physical barrier of the cornea, which restricts drug penetration in the eye, as well as the presence of eye injury that necessitates continuous delivery of growth factors on the ocular surface for cornea healing. Here, we introduce a transformative wearable corneal microneedle (MN) patch designed for efficient therapy of ocular injury and infection. The MN patch comprises water‐soluble tips that encapsulate antibacterial nanoparticles (NPs), along with a transformative backing layer that contains epidermal growth factor (EGF). Upon insertion into the eye, the MN tips dissolve swiftly within the cornea stroma, resulting in the release of the antimicrobial NPs to efficiently eradicate bacteria. Meanwhile, the residual backing layer undergoes rapid in situ transformation upon contact with mildly acidic fluid from infected corneal edema, converting into a contact lens that conforms to the eye's surface, which facilitates sustained release of EGF on the ocular surface over 8 h to promote corneal healing. Benefiting from these features, the designed transformative corneal MN patch demonstrates superior efficacy in treating ocular injuries and infections in vivo, offering a promising therapeutic strategy to manage eye diseases.

## Graphical abstract

This work describes a soft transformative microneedle (MN) patch that enables direct delivery of antimicrobial nanoparticles (NPs) into the cornea for effective bacterial elimination and allows in situ transformation to a contact lens for sustained release of epidermal growth factor (EGF) to advance cornea healing.

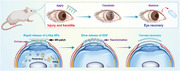

## 1 Introduction

Ocular injury is one of the most under‐recognized causes of vision loss in the world, which usually results from bruises, punctures, burns, and scratches.^[^ ^1^ ^]^ It is estimated that there are ≈55 million people worldwide experiencing eye injuries each year, creating a huge global burden.^[^ ^2^ ^]^ Additionally, the ocular injury may allow microorganisms to enter the damaged cornea, thereby causing corneal infections (i.e., bacterial keratitis),^[^ ^3^ ^]^ which further exacerbates eye conditions and poses a significant impact on patient life quality. Injury‐induced bacterial keratitis frequently leads to corneal edema, which occurs due to the impaired fluid filtration function of the endothelium and the accumulation of mildly acidic fluid within the cornea.^[^ ^4^, ^5^ ^]^

Eye drops containing antibiotics are the first‐line treatment for bacterial keratitis therapy. However, this formulation usually requires frequent administration, (e.g., hourly instillation for 1 week) due to the physical barrier of the corneal epithelium, which restricts drug diffusion, and rapid clearance by ocular fluids, which shortens drug retention time in the eye, thereby significantly reducing drug bioavailability (less than 5%) and affecting patient compliance.^[^ ^6^, ^7^ ^]^ Despite some efforts that have been made to enhance ocular absorption of the topical formulation, such as the addition of viscosity‐enhancing polymers,^[^ ^8^ ^]^ or the creation of a thin‐film on the ocular surface,^[^ ^9^ ^]^ the efficiency of drug delivery through the cornea remains unsatisfactory. Ocular implants are another traditional system for ophthalmic drug delivery and can greatly improve drug delivery efficiency and prolong release time, whereas such therapeutic method typically involves surgical procedures for implantation (biodegradable) or implantation and removal (non‐biodegradable), which can cause patient discomfort and pose risks of ocular secondary injury.^[^ ^10^, ^11^ ^]^ To address the limitations of conventional formulations, drug‐eluting contact lenses have been developed for ocular drug delivery, which can adhere to the tear film of the cornea via surface tension, thereby extending drug retention and enabling continuous drug release into the eye in a non‐invasive manner.^[^ ^12^, ^13^, ^14^ ^]^ However, drug‐encapsulated contact lenses often face challenges such as limited drug loading and diffusion through the cornea, undesired drug leakage during storage, and reduced drug stability, particularly problematic for biological macromolecules like proteins.^[^ ^15^, ^16^ ^]^ These issues significantly restrict their broader application in treating eye diseases.

Recently, microneedles (MNs) that have been extensively utilized for transdermal drug delivery across various biomedical applications such as immunization,^[^ ^17^, ^18^ ^]^ diabetes,^[^ ^19^, ^20^ ^]^ cancers,^[^ ^21^, ^22^ ^]^ wound healing,^[^ ^23^, ^24^ ^]^ and hair loss,^[^ ^25^, ^26^ ^]^ have been adopted for ocular drug delivery owing to their unique properties, including minimal invasiveness, painlessness, self‐administration capability, enhanced drug bioavailability, and improved patient compliance.^[^ ^27^, ^28^, ^29^ ^]^ For example, a dissolvable MN patch was designed for the delivery of fluconazole with improved drug penetration into the eye, which demonstrated increased effectiveness in treating fungal keratitis compared to topical dosing.^[^ ^30^ ^]^ Predatory Bacteria could be delivered into the eyes via cryoMN against eye infection, which successfully impeded the growth of gram‐negative bacteria in a rodent eye infection model.^[^ ^31^ ^]^ Our group also reported a core–shell MN patch designed for rapid implantation of biodegradable MNs into the cornea, which allows for programmed drug release, leading to the efficient elimination of bacterial infections in ocular tissue.^[^ ^32^ ^]^ Despite the progress, there remains limited work on MN patches for the therapy of ocular injury and infection, primarily due to the dual requirement of rapidly removing bacteria from the cornea and sustaining the release of growth factors on the ocular surface to promote corneal epithelial migration and proliferation.

**Figure 1:**
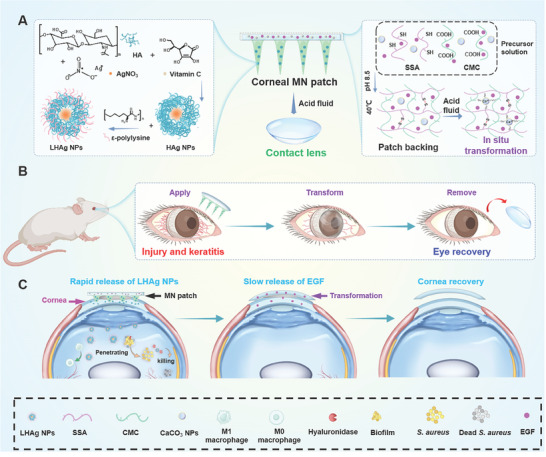
The schematic diagram of the application of the transformative corneal MN patch for ocular injury and infection treatment. A) Composition of MNs and patch backing of the corneal MN patch. B) Schematic illustration of the application of the transformative corneal MN patch in the eye. C) Schematic illustration of the working mechanism of the transformative corneal MN patches in the eye.

Building on the literature and our previous work, we describe a soft transformative MN patch that enables direct delivery of antimicrobial nanoparticles (NPs) into the cornea for effective bacterial elimination and allows in situ transformation to a contact lens for sustained release of epidermal growth factor (EGF) to advance cornea healing (**Figure** **1**A). The MN patch is designed with dissolvable MNs made of water‐soluble polyvinyl alcohol (PVA) polymer and a transformative hydrogel‐based backing layer (Figure 1B). The MNs encapsulate ε‐polylysine and hyaluronic acid (HA)‐modified silver NPs (i.e., LHAg NPs) and can directly deliver the NPs into corneas with improved delivery efficiency for antibacterial effect after cornea insertion. Simultaneously, the patch backing that is comprised of sulfhydrated sodium alginate (SSA), carboxymethyl chitosan (CMC), CaCO_3_ NPs, and EGF, shows Ca^2+^‐induced in situ gelation upon contact with the mildly acidic fluid from infected corneal edema, converting into a contact lens on the ocular surface. The formed contact lens possesses superior anti‐swelling and light‐transmittance properties and fits well under the eyelid, serving as a drug depot on the ocular surface for sustained release of EGF for more than 8 h to facilitate cornea wound healing (Figure 1C). We believe that the soft transformative MN patch, which is safe, biocompatible, and effective for ocular drug delivery, will provide a valuable option for patients to improve the management of eye diseases.

## 2 Results

### 2.1 Synthesis and Characterization of LHAg NPs

**Figure 2:**
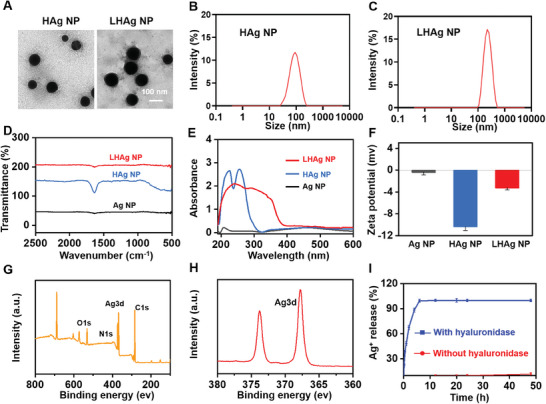
Characterization of LHAg NP. A) Representative TEM images of HAg NP and LHAg NP, respectively. Size distribution of HAg NP B) and LHAg NP C), respectively. FTIR spectra D), UV spectra E), and zeta potentials F) of Ag NP, HAg NP, and LHAg NP, respectively. G) XPS spectrum of LHAg NP. H) The divisible peaks of Ag3d in XPS of LHAg NP. I) The Ag^+^ release from LHAg NP in the medium with or without hyaluronidase at pH 6.4.

We used HA as the stabilization agent and vitamin C as the reduction agent to prepare HAg NPs, which were then modified with ε‐polylysine to synthesize LHAg NPs. Both the HAg NPs and LHAg NPs showed uniform morphology (**Figure** **2**A), with the particle size of 84.3 ± 6.1 and 108.2 ± 4.9 nm, respectively (Figure 2B,C). The successful synthesis of LHAg NPs was validated by Fourier‐transform infrared spectroscopy (FTIR) (Figure 2D) and UV–vis spectrophotometer (Figure 2E). The change of the zeta potential between HAg NPs and LHAg NPs was due to the fact that the ε‐polylysine carried a high positive charge (Figure 2F). Further, the X‐ray photoelectron spectroscopy (XPS) was used to analyze the elemental composition of the LHAg NPs, and the four main peaks at 531.5, 399.5, 367.8, and 284.4 eV corresponded to the binding energy of O1, N1, Ag3d, and C1 s, respectively (Figure 2G,H). Collectively, these results demonstrated the successful synthesis of the LHAg NPs. The release of Ag^+^ from LHAg NPs was finally detected by Inductively Coupled Plasma Mass Spectrometry (ICP‐MS) (Figure 2I), which showed a rapid release in the presence of hyaluronidase, but displaying a negligible release without hyaluronidase, suggesting the hyaluronidase‐responsive property of the LHAg NPs. The release medium with pH 6.4 was chosen because the *Staphylococcus aureus* (*S. aureus*)‐infected area is generally believed to be weakly acidic in the range of 5.0–6.5.^[^ ^33^ ^]^

### 2.2 Antibacterial effect of LHAg NPs

**Figure 3:**
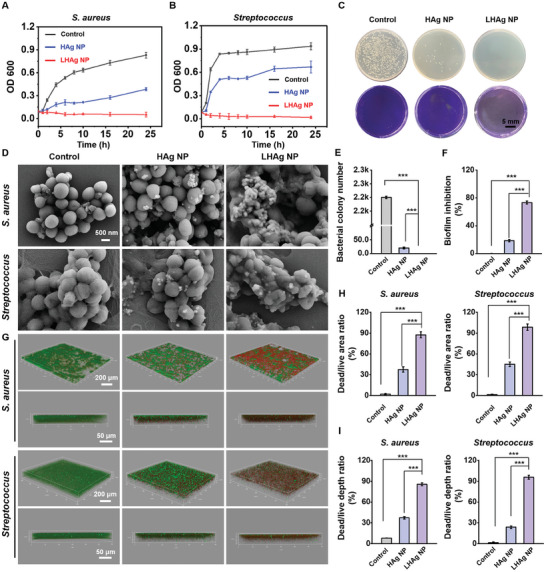
The anti‐bacterial effect of LHAg NP in vitro. The growth inhibition curve of *S. aureus* A) and *Streptococcus* B) when culturing with HAg NP or LHAg NP (*n* = 3). C) The agar plate of *S. aureus* cultured with HAg NP or LHAg NP (upper) and the crystal violet staining of *S. aureus* biofilm cultured with HAg NP or LHAg NP (lower). D) Representative SEM images of *S. aureus* and *Streptococcus* cultured with HAg NP or LHAg NP. E) The bacterial colony number of *S. aureus* in the agar plate (*n* = 3). F) The biofilm scavenge rate was analyzed in the crystal violet staining (*n* = 3). G) The SYTO9/PI staining of *S. aureus* and *Streptococcus* biofilm cultured with HAg NP or LHAg NP. H) Dead/live area ratio of bacteria after incubation with HAg NP or LHAg NP (*n* = 3). I) Dead/live depth ratio of bacteria after incubation with HAg NP or LHAg NP (*n* = 3). (^***^ *p* < 0.001).

We first investigated the antibacterial effect of LHAg NPs. As shown in **Figure** **3**A,B, LHAg NPs exhibited significant bacterial proliferation inhibition after incubation with the bacteria of *S. aureus* or *Streptococcus*, which was further confirmed by the scanning electron microscope (SEM) result that showed significant destruction on bacterial cell membrane and caused intracellular leakage (Figure 3D). It was determined that the minimum inhibitory concentration of LHAg NPs on *S. aureus* or *Streptococcus* was ≈8 and 20 µg mL^−1^, respectively (Figure S1, Supporting Information). The single colony plating and crystal violet staining (Figure 3C) of *S. aureus* showed that the LHAg NPs significantly restricted bacterial growth (Figure 3E) and inhibited the biofilm formation (Figure 3F). The antimicrobial activity of NPs was further validated by their ability to inhibit the growth of drug‐resistant *S. aureus* (Figure S2, Supporting Information) and disrupt biofilm formation (Figure S3, Supporting Information). Such superior antibacterial features of LHAg NPs were probably due to the increased penetration of the NPs in the adhesive and thick biofilm, which was validated by the SYTO9/PI staining outcomes (Figure 3G–I).

### 2.3 The Formation of CSSA/Ca Hydrogel

**Figure 4:**
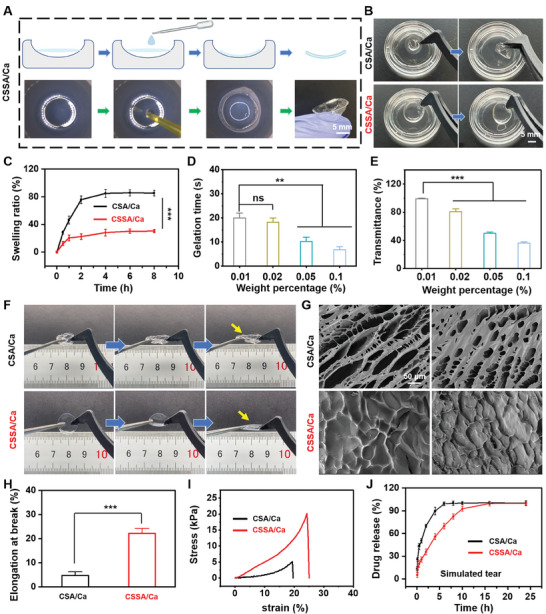
The characterization of CSSA/Ca hydrogel. A) The transformation of CSSA/Ca hydrogel to a contact lens in response to acid environment (pH 6.4). B) The swelling test of CSA/Ca (upper) and CSSA/Ca (lower) in tear simulated buffer solution. C) The swelling ratio of CSA/Ca and CSSA/Ca hydrogel (*n* = 3). D) The gelation time of CSSA/Ca hydrogel containing different concentrations of CaCO_3_ (0.01%, 0.02%, 0.05%, or 0.1%) (*n* = 3). E) The transmittance of CSSA/Ca hydrogel containing different concentrations of CaCO_3_ (0.01%, 0.02%, 0.05%, or 0.1%) (*n* = 3). F) The tensile strength test of CSA/Ca and CSSA/Ca hydrogel. The yellow arrowheads indicate the position of a hydrogel. G) Representative SEM images of CSA/Ca and CSSA/Ca hydrogel. H) The elongation rate of CSA/Ca or CSSA/Ca hydrogel (*n* = 3). I) The compressive stress/strain curve of CSA/Ca and CSSA/Ca hydrogel. J) The release profile of FITC‐BSA from CSA/Ca or CSSA/Ca hydrogel in vitro (*n* = 3). All data are represented as mean ± SD. (^**^ *p* < 0.01, ^***^ *p* < 0.001). The ns indicates no significance.

The backing layer of the corneal MN patch was comprised of CMC, SSA, CaCO_3_ NPs, and EGF. The CaCO_3_ NPs were synthesized through volatilization in a vacuum drying oven for 24 h. The particle size of CaCO_3_ NPs was ≈147.6 ± 3.2 nm, which was measured by dynamic light scattering (DLS). The NPs possessed pH‐responsive characteristics, as evidenced by the fast release of abundant Ca^2+^ and obvious morphology change (Figure S4, Supporting Information) within a short time in the medium at pH 6.4. Encouraging by this, we hypothesize that CaCO_3_ NPs can be used as the Ca^2+^ source to trigger the crosslink of SSA and CMC in a weakly acidic environment, thereby causing in situ transformation of MN patch backing. As shown in **Figure** **4**A, the thin‐film dried from the mixture of CMC, SSA, and CaCO_3_ NPs (CSSA/Ca), rapidly changed to soft hydrogel after contact with a mildly acidic PBS solution (pH 6.4), exhibiting an intact curvature, similar to a contact lens. We also exposed the backing film to environments with different pH values (5.0, 5.5, and 6.0). As shown in Figure S5 (Supporting Information), the backing film rapidly converted into a hydrogel at all tested pH values, exhibiting excellent transmittance, which suggests promising translational potential for the device. Importantly, the CSSA/Ca hydrogel exhibited superior anti‐swelling property after being immersed in tear‐simulated buffer solution for 24 h, maintaining its morphology over time, in comparison to CSA/Ca (consisting of CMC, SA, and CaCO_3_ NPs) hydrogel that showed a broken shape due to excessive swelling (Figure 4B). Such an anti‐swelling feature was further verified by the measurement of the swelling ratio of the CSSA/Ca hydrogel after buffer solution soaking for 8 h, which was significantly smaller than that of the CSA/Ca hydrogel (Figure 4C).

To select the optimal CaCO_3_ NPs concentration, the gelation time and light transmission of the gel were investigated. The percentages of 0.01% and 0.02% CaCO_3_ NPs required the most time for transformation compared to 0.05% and 0.1% CaCO_3_ NPs (Figure 4D). However, 0.01% CaCO_3_ NPs exhibited the highest transmittance among all concentrations (Figure 4E; Figure S6A, Supporting Information). There was no significant difference in the drug release profiles among the different concentrations of CaCO_3_ NPs (Figure S6B, Supporting Information), and the CSSA/Ca hydrogel with varying percentages of CaCO_3_ NPs exhibited good biocompatibility without causing apparent hemolysis (Figure S6C, Supporting Information). As a result, the concentration of 0.01% CaCO_3_ NPs was selected in the following experiments. Notably, the transmittance of the CSSA/Ca hydrogel did not exhibit any significant change after immersion in tear‐simulated buffer for 72 h in vitro, nor after prolonged use on rat corneas in vivo (Figure S7, Supporting Information). Additionally, the oxygen permeability of the hydrogel patch was measured to be 28.1 ± 0.3 Dk/t, which is adequate for a healthy oxygen supply to the cornea during normal daily wear.^[^ ^34^ ^]^

We next examined the elasticity and toughness of the CSSA/Ca hydrogel, and found that it exhibited a significantly higher elongation rate compared to the CSA/Ca hydrogel (Figure 4F,H). In addition, the stress–strain curve showed that the CSSA/Ca hydrogel could withstand higher deformation in comparison to CSA/Ca hydrogel, and could be destroyed within the stress approaching 20 kPa, demonstrating the good elasticity of the CSSA/Ca hydrogel (Figure 4I). Finally, the model drug of bovine serum albumin (BSA) was loaded in hydrogels, which was used to predict drug release from the hydrogels. As shown in Figure 4J, the CSSA/Ca hydrogel demonstrated much slower drug release compared to the CSA/Ca hydrogel, probably due to the intact structure of the CSSA/Ca gel surface, which was evidenced by the SEM images of the hydrogel morphology (Figure 4G).

### 2.4 In Situ Transformation of the Corneal MN Patch to a Contact Lens

**Figure 5:**
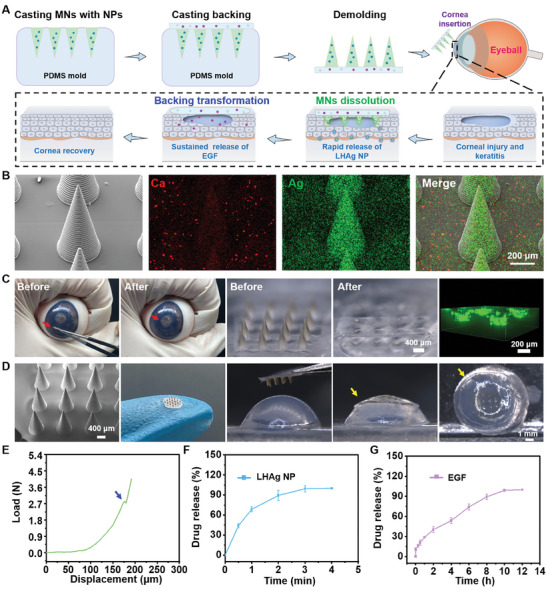
Characterization of the corneal MN patch in vitro. A) The fabrication and working process of corneal MN patches. B) Representative SEM image and energy‐dispersive spectroscopy (EDS) elemental mapping analysis of corneal MNs. C) Representative bright images of the corneal MN patch before and after insertion in a porcine eye ex vivo, and a representative fluorescent 3D image showing the penetration of MNs in the porcine eyeball. The red arrowhead indicates the corneal MN patch. D) Insertion and transformation of the corneal MN patch on an agarose‐simulated eyeball model in response to the acid condition (pH 6.4). The yellow arrowhead indicates the position of the hydrogel‐based contact lens transformed from patch backing. E) Mechanical test of the corneal MN patch. F) The release of LHAg NPs from the corneal MN patch in vitro (*n* = 3). G) The release of EGF from the corneal MN patch in vitro (*n* = 3).

The corneal MN patch was fabricated using a sequential casting method. The polymer of PVA containing LHAg NPs was first cast in a polydimethylsiloxane (PDMS) mold to form the MNs. Subsequently, the mixture of CMC, SSA, CaCO_3_ NPs, and EGF was added to the top of the mold to form the backing layer of MN patches (**Figure** **5**A). The MN patch was confirmed by the energy‐dispersive spectroscopy (EDS) elemental mapping analysis (Figure 5B), which showed that Ag element mostly accumulated in MNs and Ca element widely distributed in the backing part. The resulting corneal MN patch comprised a 4 × 4 array of MNs, with each MN 850 µm in height. The MN patch possessed strong mechanical strength (Figure 5E), and could successfully insert into the porcine eyeball ex vivo, leaving no needles left in the residual patch (Figure 5C). The successful penetration of the corneal MN patch in the porcine eyeball was confirmed by the 3D scanning image (right panel in Figure 5C). Such a result demonstrated the feasibility of the corneal MN patch for ocular drug delivery in large animal species. To further examine the in situ transformation of the MN patch into a contact lens, the MN patch was next applied to agarose (pH 6.4), which showed that the MNs rapidly dissolved and the backing layer quickly converted into a transparent hydrogel that conformed to the curvature of the hemispheres, demonstrating excellent shape adaptability of the patch backing (Figure 5D). Next, LHAg NPs release from the corneal MN patch was evaluated in vitro using agarose gel, and EGF release was tested in the simulated tear, respectively. The result showed that the antibacterial LHAg NPs were released within ≈3 min (Figure 5F), while EGF release from the backing layer extended up to 10 h (Figure 5G), indicating a programmed drug release property of the corneal MN patches. Due to the rapid dissolution of MNs and fast release of LHAg NPs, the corneal MN patch exhibited an excellent antibacterial effect in vitro (Figure S8, Supporting Information), which was demonstrated by the SYTO9/PI staining and the antibacterial zone experiment.

### 2.5 Promotion on Cell Migration, Proliferation, and Adhesion by Corneal MN Patches In Vitro

**Figure 6:**
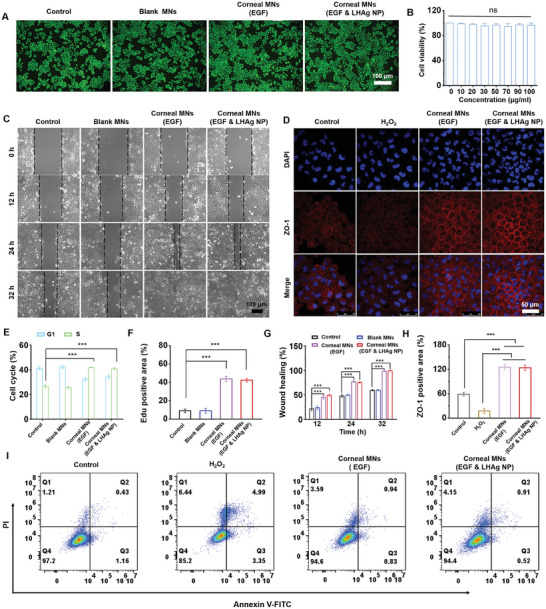
Effect of the corneal MN patch on cell migration, proliferation, and adhesion in vitro. A) Representative Calcein/PI staining images of HCEC after receiving different treatments. B) Cell viability of HCEC after incubation with different concentrations of LHAg NP (0, 10, 20, 30, 50, 70, 90 and 100 µg mL^−1^). C) Representative photographs of HCEC scratches at different time points (0, 12, 24 and 32 h) after incubation with different MN patches. D) Representative ZO‐1 immunofluorescence images of HCEC incubated with different MN patches after H_2_O_2_ injury. E) The G1 and S cell cycle percentage of HCEC incubated with different MN patches. F) The Edu positive area of HCEC incubated with different MN patches. G) Wound healing rate in the scratch experiment. H) The ZO‐1 positive area in the immunofluorescence experiment. I) The cell apoptosis of HCEC incubated with different MN patches after H_2_O_2_ injury. All data are represented as mean ± SD. (^***^ *p* < 0.001). The ns indicates no significance.

The corneal MN patch displayed good biocompatibility with human corneal endothelial cells (HCECs), causing negligible cell apoptosis even at a concentration of 100 µg Ml^−1^ of LHAg NPs (**Figure** **6**A,B). We also examined cell migration behavior by performing scratch assays on HCECs treated with different corneal MN patches. Addition of EGF significantly increased cell migration rates, which was observed in both groups of corneal MNs (EGF) and corneal MNs (EGF & LHAg NPs) (Figure 6C,G). We also found that the treatment with corneal MNs (EGF) or corneal MNs (EGF & LHAg NPs) drove more cells to enter the S phase (Figure 6E; Figure S9, Supporting Information), that is, the DNA synthesis phase, as confirmed by the Edu staining result (Figure 6F; Figure S10, Supporting Information), suggesting that addition of EGF could promote cell proliferation. To assess whether corneal MN patches provide protective effects on cell adhesion, the ZO‐1 expression, a scaffolding protein crucial for tight junction functions and forming the primary barrier in corneal epithelial cells, was analyzed via immunofluorescence, which showed significant elevation of ZO‐1 expression with corneal MN patches compared to the control group (Figure 6D,H). Moreover, treatment with corneal MN patches significantly reduced cell necrosis rates (Figure 6I), further validating their capability to promote corneal epithelial rebuilding in bacterial keratitis therapy.

### 2.6 Regulation of Corneal MN Patches on Macrophage and Inflammation In Vitro

**Figure 7:**
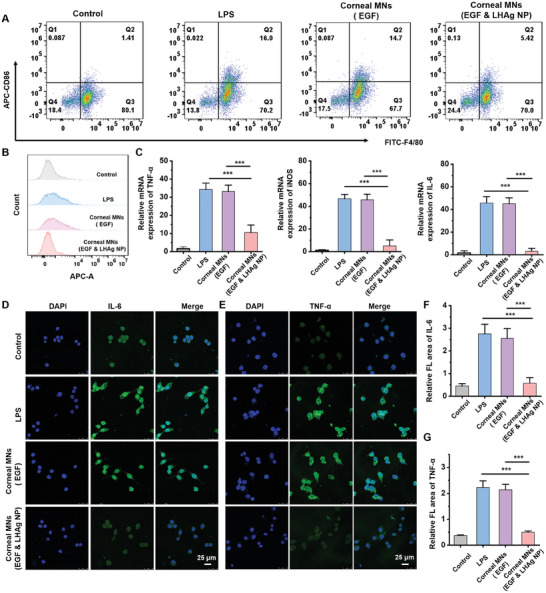
Regulation and inhibition of corneal MN patches on inflammation in vitro. A) The CD86(+)/F4/80(+) cell population incubated with different corneal MN patches under LPS‐induced situation. B) Flow cytometry histogram of CD86 expression in RAW 264.7 cells. C) The mRNA expression of TNF‐α, iNOS, and IL‐6 in RAW 264.7 cells after incubation with corneal MN patches under LPS‐induced situation (*n* = 3). The immunofluorescence of IL‐6 D) and TNF‐α E) in RAW 264.7 cells after incubation with different corneal MN patches under LPS‐induced situation. The statistical chart of relative fluorescence (FL) area of IL‐6 F) and TNF‐α G) (*n* = 3). All data are represented as mean ± SD. (^***^ *p* < 0.001).

We next studied the anti‐inflammatory effect of corneal MN patches. Lipopolysaccharide (LPS)‐induced RAW 264.7 cells typically upregulate the expression of CD86. However, after incubation with the corneal MN patch, the production of CD86 was greatly reduced in LPS‐treated RAW 264.7 cells, indicating a notable anti‐inflammatory effect of the corneal MN patch (**Figure** **7**,B). This anti‐inflammatory property was further validated by real‐time quantitative polymerase chain reaction (qPCR) results, which determined a significant decrease in the mRNA expression levels of tumor necrosis factor‐alpha (TNF‐α), inducible nitric oxide synthase (iNOS), and interleukin‐6 (IL‐6) in RAW 264.7 cells treated with corneal MN patches (Figure 7C). Additionally, immunofluorescence analysis of IL‐6 (Figure 7D,F) and TNF‐α (Figure 7E,G) further confirmed that the corneal MN patches effectively inhibited M1 polarization and significantly reduced the levels of these inflammatory cytokines.

### 2.7 Promotion of Corneal MN Patches in the Healing of Eye Scratches In Vivo

**Figure 8:**
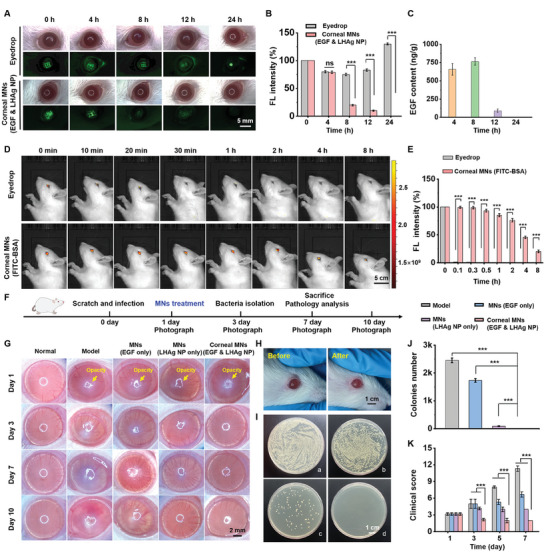
Application of corneal MN patches in vivo. A) The healing process of the scratch‐induced wound after application of corneal MN patches in rats, as indicated by fluorescein sodium staining. B) The fluorescence (FL) intensity in (A) (*n* = 5). C) The EGF content in the cornea at different time points after corneal MN patches (EGF & LHAg NP) treatment (*n* = 5). D) The images of rat eyes in vivo after the treatment with FITC‐BSA eyedrop and the corneal MN patch (containing the same FITC‐BSA). E) The FL intensity of the eyedrop group and corneal MN patch group in the rat eyes over time (*n* = 5). F) Schematic diagram of the model establishment and treatment process of the ocular injury and infection. G) Representative photographs of rat eyes in different groups. The eye of a healthy rat was used as a positive control (normal group), and the eye of a keratitis rat without any treatment was recognized as a negative control (model group). H) Representative photographs of rat eyeball in vivo before and after the corneal MN patch application. I) The agar plate culture of *S. aureus* isolated from of rat eyeballs in the groups of model (a), corneal MNs (EGF) (b), corneal MNs (LHAg NP) (c), and corneal MNs (EGF & LHAg NP) (d), respectively. J) The colonies number in the agar plate of isolated *S. aureus* in each group (*n* = 5). K) The clinical score of rats in each group (*n* = 5). All data are represented as mean ± SD. (^***^ *p* < 0.001).

The corneal MN patch demonstrated effective penetration in rat corneas in vivo, with a puncture depth of ≈70 µm (Figure S11, Supporting Information), largely due to the abundant fluid on the ocular surface and within the cornea. To evaluate the effect of the MN patch on corneal wound healing in vivo, the corneal MN patch was applied to the ocular surface following a scratch operation. Fluorescein sodium was then used to monitor the healing process. The staining result revealed that the corneal scratch gradually healed and the fluorescence that represented the existence of the wound completely disappeared 24 h post the application of the corneal MN patch, whereas the stained scratches could still be observed within 24 h after the use of eyedrops (**Figure** **8**A,B), indicating the promotion effect of the corneal MN patch on corneal scratch healing. Although the insertion of MNs created microchannels in the eyes, these channels closed rapidly after 8 h (Figure S12, Supporting Information), thereby eliminating the potential risk of bacterial infection. The results of H&E staining (Figure S13, Supporting Information) and optical coherence tomography (OCT) (Figure S14, Supporting Information) further demonstrated the efficacy and biocompatibility of the MN patch in treating ocular injury and infection in vivo.

We further investigated the drug retention and sustained release properties of the corneal MN patch. For better visualization, fluorescein isothiocyanate (FITC)‐conjugated BSA (FITC‐BSA) served as a model drug to simulate the drug retention of the corneal MN patch. As shown in Figure 8D,E, the fluorescent model drug remained visible in the rat eye for over 8 h when wearing the corneal MN patch, whereas the drug was rapidly cleared and no longer detectable 10 min after eyedrop administration, indicating a significant improvement in drug retention on the ocular surface facilitated by the corneal MN patch. The prolonged drug retention feature supported sustained drug release in ocular tissues, as evidenced by the release profile of EGF from the wearable corneal MN patch in the eyes for 8 h (Figure 8C), suggesting the long‐term drug retention and release capabilities of the corneal MN patches.

### 2.8 Therapeutic Efficacy of Corneal MN Patches for Scratch‐Induced Keratitis In Vivo

To examine the therapeutic efficacy of the corneal MN patch in vivo, injury‐induced keratitis was first established in rats through scratching the eye epithelium and inoculating *S. aureus* to infect the injured tissue (Figure 8F). One day after the operation, the eyes exhibited opacification (Figure 8G), indicating the successful establishment of injury‐induced bacterial keratitis. The rats were then randomly divided into four groups, receiving: 1) no treatment (i.e., model group), 2) corneal MN patches with only EGF, 3) corneal MN patches with only LHAg NPs, and 4) corneal MN patches with both EGF and LHAg NPs. Healthy rats without keratitis were used as a negative control (i.e., normal group). The morphology of eyeballs was recorded on days 1, 3, 7, and 10. It was observed that rats that received the treatment of the corneal MN patch with LHAg NPs and EGF, exhibited reduced opacity and limited neovascularization in the eye compared to other groups, suggesting superior therapeutic efficacy in treating bacterial keratitis (Figure 8G,H).

**Figure 9:**
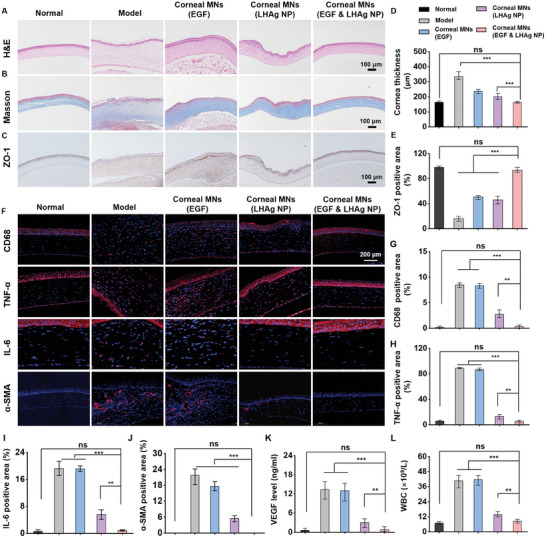
Pathologic analysis of cornea after corneal MN patches treatment. A) The staining of H&E (A), Masson B), and ZO‐1 C) of rat corneas 7 days after receiving different treatments. D) The cornea thickness of rats in each group 7 days post treatments (*n* = 5). E) The ZO‐1 positive area in each group (*n* = 5). F) The CD68, TNF‐α, IL‐6, and α‐SMA immunofluorescence images of rat corneas in each group. The positive area of CD68 G), TNF‐α H), IL‐6 I), and α‐SMA J) in the immunofluorescence staining result (*n* = 5). K) VEGF level in the cornea in each group (*n* = 5). L) The WBC level of blood in rats in each group (*n* = 5). All data are represented as mean ± SD. (^**^ *p*< 0.01, ^***^p < 0.001). The ns indicates no significance.

To further investigate the antibacterial effect of the corneal MN patch in vivo, bacterial samples were collected from the cornea on day 3 and cultured on agar plates, which showed that *S. aureus* growth was significantly inhibited after receiving the treatment of the corneal MN patch containing LHAg NPs and EGF (Figure 8I,J). This finding was consistent with the morphological observation of rat eyes (Figure 8G). We also collected rat eyeballs and determined the LHAg NPs concentration as 38.5 ± 3.9 µg mL^−1^ in the cornea, which was greater than the minimal inhibitory concentration of the NPs, further confirming the effective anti‐bacterial activity of the corneal MN patch in vivo. The delivered NPs could achieve the release of Ag ions in the cornea for over 12 h (Figure S15, Supporting Information). Clinical scores for rats in each group were assessed based on opacification, ulceration, and vascular proliferation. A higher clinical score indicated more severe keratitis. As shown in Figure 8K, the corneal MN patch with LHAg NPs and EGF achieved the lowest clinical score on days 3, 5, or 7, reflecting the most effective therapeutic outcome for keratitis treatment. Such superior therapeutic effect of the corneal MN patch was further confirmed by histological analysis, including H&E staining (**Figure** **9**A,D), Masson staining (Figure 9B), and immunohistochemical staining (Figure 9C,E), which revealed excellent corneal recovery, characterized by a well‐formed corneal epithelium and a clear matrix layer. Furthermore, immunofluorescence staining of transforming growth factor‐*β* (TGF‐*β*), which plays a central role in corneal wound healing after ocular injury, revealed that treatment with the corneal MN patch significantly increased TGF‐*β* expression in the corneal stroma (Figure S16, Supporting Information), demonstrating the effect of the MN patch on the protein level of corneal stromal cells in ocular injury therapy.

### 2.9 Anti‐Inflammatory Effect of Corneal MN Patches In Vivo

The anti‐inflammatory effect of corneal MN patches was finally evaluated through the analysis of CD68, TNF‐α, IL‐6, and alpha‐smooth muscle actin (α‐SMA) expression in vivo (Figure 9F). The results indicated that levels of CD68 (Figure 9G), TNF‐α (Figure 9H), and IL‐6 (Figure 9I) were significantly elevated in the model group due to bacterial invasion and subsequent inflammation. In contrast, the application of the corneal MN patch effectively reduced the expression of these inflammatory markers, demonstrating its significant anti‐inflammatory effect. New blood vessels typically form and extend into the cornea following pathogenic invasion, driven by inflammatory mediators and impaired limbal barrier function. To better understand corneal wound healing after keratitis treatment, we examined α‐SMA expression, a marker of blood vessels. As shown in Figure 9F,J,K, the corneal MN patch containing LHAg NPs and EGF exhibited fewer α‐SMA positive areas and lower levels of vascular endothelial growth factor (VEGF) compared to other treatments, suggesting a reduced growth of new blood vessels in the cornea, which further demonstrates the anti‐inflammatory efficacy of the corneal MN patch. Additionally, white blood cells (WBC) returned to the normal level after the treatment with the corneal MN patch (Figure 9L), indicating effective recovery from the ocular disease. Importantly, no significant changes were observed in the organs (heart, liver, spleen, lung, and kidney) 10 days after application of the corneal MN patch (Figure S17, Supporting Information), underscoring its satisfactory biosafety in vivo.

## 3 Discussion

Due to the physical barrier of the cornea and the need for sustained release of growth factors on the ocular surface, topical formulations often exhibit low drug bioavailability and poor therapeutic efficacy in treating ocular injuries and infections.^[^ ^35^ ^]^ Although drug‐eluting contact lenses have made great advancements in extending drug retention on the eye surface, they still face challenges such as limited drug penetration into the cornea, undesired drug leakage during storage, and reduced drug stability. MN patches represent a novel transdermal drug delivery system with enhanced delivery efficiency and improved patient compliance, and have been recently adopted for ophthalmic drug delivery for eye disease treatment. Our previous work has demonstrated the feasibility of ocular MN patches in efficiently treating eye infections through the delivery of both antibacterial and anti‐inflammatory drugs in the cornea.^[^ ^32^ ^]^ However, there is still limited research on the use of MN patches for the therapy of eye injuries and associated infections.

Building on previous literature and our prior work, we design a transformative wearable corneal MN patch with programmed drug release for efficient therapy of ocular injury and infection in this work. The MN patch is composed of water‐soluble MNs and a hydrogel‐based backing layer. Upon insertion into the cornea, the MNs dissolve rapidly, releasing encapsulated antibacterial NPs to effectively eliminate harmful bacteria. Simultaneously, the patch backing undergoes rapid Ca^2+^‐triggered in situ gelation in response to mildly acid fluid, thereby converting into a soft contact lens to conform to the curvature of the eye, which subsequently serves as a drug depot on the ocular surface for continuous release of EGF for 8 h to promote corneal wound healing. Therefore, the developed ocular MN patch that not only enhances drug delivery efficiency through the cornea but also achieves sustained release of growth factors on the eye surface, offers a promising alternative for patients suffering from ocular injuries and infections.

To eliminate deleterious bacteria from tissues, AgNPs are an excellent antimicrobial agent due to their broad spectrum of antibacterial activity and satisfactory biocompatibility.^[^ ^36^, ^37^ ^]^ Building on the advantages of AgNPs, we innovatively utilized HA to stabilize them, and further modified them with ε‐polylysine to enhance their capability to penetrate and disrupt bacterial biofilms. This enhancement was validated by the penetration result of LHAg NPs in vitro (Figure 3G–I). The antibacterial NPs and growth factors (i.e., EGF) were encapsulated in the dissolvable MNs and a hydrogel‐based backing layer, respectively, enabling dual‐release profiles. Such design facilitated the recovery from scratch‐induced injuries and infections in the eyes. Unlike eye drops or drug‐eluting contact lenses, which rely on drug diffusion through the cornea for ocular drug delivery, the transformative ocular MN patch not only increases drug delivery efficiency by directly administering antibacterial agents from the dissolvable MNs, but also supports the sustained release of EGF from the transformed contact lens on the ocular surface to promote ocular wound healing. Additionally, the MN patch offers superior potential for preventing drug leakage and improving drug stability due to its solid formulation during storage. Importantly, the contact lens derived from the transformative MN patch exhibits exceptional anti‐swelling and light‐transmitting properties, which is very crucial for the improvement of patient compliance.^[^ ^38^ ^]^

In summary, the developed MN patch provides a safe and effective option for patients to improve the management of eye injuries and infections. Future studies investigating the ocular MN patch in larger animal species, such as rabbits or pigs, along with an assessment of potential risks, including tear film instability and the release of excessive Ca^2+^ into the eyes after application, will significantly advance this research toward potential future applications in public health.

## 4 Experimental Section

### Materials

Calcium chloride (CaCl_2_), Ammonium bicarbonate (NH_4_HCO_3_)_,_ Silver nitrate (AgNO_3_), 5‐aminofluorescein, 5,5′ ‐disulfide (2‐nitrobenzoic acid) (DTNB) were purchased from Macklin (Shanghai, China). Hyaluronic acid (HA), SA, 1‐(3‐dimethylaminopropyl) ‐3‐ethylcarbodiimide (EDC), n‐hydroxysuccinimide (NHS), BSA, FITC, and fluorescein sodium were purchased from Aladdin (Shanghai, China). Polydimethylsiloxane (PDMS) was purchased from Dow Corning (Midland, USA). Ca^2+^ concentration detection kit, Calcein AM/PI kit, MTT, H&E kit, and Masson kit were purchased from Beyotime (Shanghai, China). DAPI and SYTO9/PI kits were purchased from MKbio (Shanghai, China). EGF ELISA kit, VEGF ELISA kit, and Annexin V‐FITC/PI kit were purchased from Procella (Wuhan, China). Hifair II 1st Strand cDNA Synthesis SuperMix and Hieff qPCR SYBR Green Master Mix were purchased from YEASEN (Shanghai, China). Hyaluronidase was purchased from Merck (New Jersey, USA). Flowjo antibody F4/80 and CD86 were purchased from Elabscience (Wuhan, China). TNF‐α and IL‐6 antibodies were purchased from Wanleibio (Shenyang, China).

### Preparation of CaCO_3_ NPs

Fifty milligram CaCl_2_ was dissolved in 200 µL water and then dispersed in 50 mL ethyl alcohol. After that, CaCl_2_ solution and 500 mg NH_4_HCO_3_ were placed in the vacuum drier at room‐temperature (RT) for 24 h. The synthesized CaCO_3_ NPs were obtained by centrifugation at 10 000 rpm and washing through ethyl alcohol three times. Finally, CaCO_3_ NPs were dried at 42 °C and then stored in a dryer.

### Preparation of LHAg NPs

First, 16 mg AgNO_3_ and 100 mg HA were dissolved in 100 mL distilled water and stirred for 2 h at RT. Then, 70 mg vitamin C was dissolved in 10 mL distilled water and then slowly added in the mixture of HA and AgNO_3_ for continuously stirring for 1 h. HAg NPs were then collected through centrifugation at 10 000 rpm for 15 min and washed through PBS for three times. After that, 10 mg HAg NPs were redispersed in 100 mL distilled water and incubated with 10 mL 0.1% ε‐polylysine solution for 2 h agitation to form LHAg NPs, which were then purified through 10 000 rpm centrifugation for 15 min and washing through PBS for three times. The release of Ag^+^ from LHAg NPs in the presence of hyaluronidase was performed in 10 mL PBS solution (pH 6.4) containing 0.5 mg mL^−1^ hyaluronidase. At different time point (0, 1, 2, 4, 6, 8, 10, 16, and 24 h), 500 µL sample was collected and simultaneously replaced with 500 µL fresh release medium, and Ag^+^ concentration was detected through ICP‐MS (Analytik Jena AG, PQ‐MS).

### Synthesis of Sulfhydrated Sodium Alginate (SSA)

To prepare SSA, 0.1 g SA was first dissolved in 10 mL water to form 1% SA solution. Next, 78.21 mg EDC and 54.23 mg NHS were used as activator agents to activate the carboxyl in the SA. This activation process was continued under RT for 2 h, after which 56 mg cysteine was added and stirred for another 12 h to finish the condensation reaction. Unreacted cysteine and activator agents were removed through dialysis for 3 days in a 3.5 kDa dialysis tube. SSA was collected via lyophilization and finally stored at 4 °C away from light.

### In Situ Transformation and Characterization of CSSA/Ca Hydrogel

The in situ transformation of CSSA/Ca‐based film was observed in the hemispherical mold. The photographs before and after dropping the pH 6.4 buffer were recorded under a stereomicroscope (Nikon, SMZ745). The transmittance of CSSA/Ca film was detected through UV–vis spectra. For the swelling rate, CSSA/Ca hydrogel was immersed in a tear‐simulated solution that was prepared according to the method described in the literature (NaCl 0.68, NaHCO_3_ 0.22, KCl 0.14, CaCl_2_·2H_2_O 0.008 g, 100 mL distilled water, pH 7.4). ^[^ ^39^ ^]^ At the time points of 1, 2, 4, 6, and 8 h, CSSA/Ca hydrogel was taken out and weighted after removing excess water by absorbent paper. The original weight was recorded as W_0_, and the weight at different time points was recorded as W_1_. The swelling rate was calculated by using the equation: swelling rate = (W_1 –_ W_0_) / W_0_ × 100%.

The tensile mechanical property of the CSSA/Ca hydrogel was tested through the manual drawing under the indicator of steel rule. The original length was recorded as L_0_, and the maximum length after deformation was recorded as L_1_. Elongation at the breakpoint was calculated by the following equation: Elongation at the breakpoint = (L_1 –_ L_0_) / L_0_ * 100%.

### Fabrication and Characterization of Cornea MN Patches

The corneal MN patches were fabricated through the layer‐by‐layer casting method. First, 50 µL of the mixture of 18% PVA solution and 1% LHAg NPs suspension was casted on a PDMS mold. After that, the casted mold was dried in a vacuum oven at 40 °C overnight. Next, 20 µL mixture (pH 8.5) of 8% CMC, 10% SSA, 0.01% CaCO_3_ NPs, and 100 µg mL^−1^ EGF was added on the PDMS mold to form the backing. The corneal MN patch was carefully demolded from the mold after desiccation under RT overnight. The mechanical property of the corneal MN patch was tested by a compression instrument (Force Gauge, Mark‐10). The drug release from the corneal MN patch was performed in a penicillin bottle. The released LHAg NPs were detected through centrifugation and weighing, and the release of EGF was measured through the ELISA kit.

### Cell Culture

Human Corneal Epithelial Cells (HCEC) were obtained from the Eye Hospital of Wenzhou Medical University. Cells were cultured with Dulbecco's Modified Eagle Medium/Nutrient Mixture F‐12 (DMEM/F12) with 10% Fetal Bovine Serum (FBS), 100 µg mL^−1^ streptomycin, and 100 U mL^−1^ penicillin, in 37 °C incubator with 5% CO_2_. Cell viability of HCEC was tested by MTT assay and Calcein‐AM/ Propidium Iodide (PI) kit. RAW 264.7 cells were obtained from the RSBM Charity cell bank. Cells were cultured with DMEM with 10% FBS in 37 °C incubator with 5% CO_2_.

### Cell Scratch Test

HCEC cells were seeded at a density of 5000 cells per well in a six‐well plate, and then incubated for 24 h. After that, the cells were scratched vertically via a sterilized yellow tip and changed with fresh medium with sterilized MNs. At different time points (0, 12, 24, and 32 h), the cell plate was observed and photographed under the microscope. The original length of the wound was L_0_, and the post‐migratory length of the wound was L_1_. The wound healing rate was analyzed by using the formula: (L_1 –_ L_0_) / L_0 _× 100%.

### The Immunofluorescence of HCEC

HCEC were incubated into the six‐well plate with a cover slide at a density of 20 000 per well. The cells were incubated with 200 µm H_2_O_2_ for 6 h except for the control group. When the cells nearly grew into a single layer, a corneal MN patch was added for 24 h incubation. After that, cell slides were fixed with 75% ethanol for 15 min, followed by embathing with PBS for three times with 3 min each time. Slides were recovered with 5% FBS at RT for 1 h. After that, slides were added with diluted primary antibody solution (ZO‐1, 1:1000) in a wet box for incubation at 4 °C overnight. The next day, the secondary antibody was dropped on the slides for incubation at 37 °C for 1 h after PBS washing. Slides were stained with DAPI for 30 min after PBS washing, and finally sealed with anti‐fluorescence quenching sealing agent. The images were observed under confocal microscopy. ZO‐1 positive fields were measured through Image J.

### CD86(+)/F4/80(+) cell Population Detection

RAW 264.7 cells were inoculated into a six‐well plate at a density of 30 000 per well and followed with 100 ng mL^−1^ LPS treatment for 12 h. The group without LPS treatment was served as the control. MNs group was given the sterilized MNs for another 24 h incubation. Next, cells were collected and then stained with CD86, F4/80 antibody at RT for 30 min. The CD86(+)/F4/80(+) cell population was detected through a flow cytometer (Beckman, CytoFLEX S). The analysis was conducted through Flow Jo 10 software.

*RT‐qPCR*: RAW 264.7 cells with a density of 30 000 cells/ well were seeded in the six‐well plate and cultured with medium containing 100 ng mL^−1^ LPS for 12 h. The group without LPS treatment was served as the control. MNs group was given the sterilized MNs for another 24 h incubation. Total RNA was extracted from cultured cells using Trizol Reagent (Invitrogen). DNA was synthesized from RNA through cDNA synthesis SuperMix (shanghai, YEASEN). And then RT‐qPCR was performed through a One‐step process by qPCR SYBR Green Master Mix following the manufacturer's protocol.

### The wound Healing of Corneal Epithelium on Rats In Vivo

After abdominal anesthesia by 2% anaobarbital sodium and surface anesthesia of 1% lidocaine, the right eye of each rat was scratched 3 mm × 3 mm matts wound with an ophthalmic scalpel in the epithelial layer of the cornea, followed with 10 µL bacterial infection (1 × 10^7^ CFU). MNs group were administrated the blank corneal MNs or corneal MNs (EGF & LHAg NPs) after the scratch wound, while PBS treatment was recognized as the control group. At different times, rats were recorded the eye and scratch wound under sodium fluorescein. The micropore closure created by MNs was simultaneously observed. After sacrifice, the cornea of rats receiving corneal MNs (EGF & LHAg NP) was collected and fully ground in distilled water. The supernatant was obtained by centrifugation to measure EGF concentration.

### Drug Retention of the Corneal MN Patch In Vivo

To investigate drug retention of the wearable corneal MN patch in vivo, FITC‐BSA, a model drug, was mixed in the CSSA/Ca‐based backing in the corneal MNs. The FITC‐BSA solution was dropped as the control. After administration with corneal MNs, the treated rat was photographed under a small animal living image (PerkinElmer, IVIS Lumina XRMS Series III) at the time points of 0, 10, 20, 30 min, 1, 2, 4, and 8 h, respectively.

### Therapy Efficacy of the Corneal MN Patches for Keratitis Treatment In Vivo

All the animal studies followed the guidelines of the Institutional Animal Care and Use Committee of Wuhan University and were approved by the Animal Ethics Committee of Wuhan University (WP20230333). The Sprague Dawley (SD) male rats (≈ 220 g) were purchased from the China Three Gorges University. The rats were fed in the alternating 12/12 h light/dark environment, with 25 ± 2 °C and 46 ± 2% humidity with unrestricted access to food and water. The animal experiment included normal, model, corneal MNs (EGF), corneal MNs (LHAg NPs), and corneal MNs (EGF & LHAg NPs) groups. Rats were anesthetized by 2% anaobarbital sodium (40 mg kg^−1^). 1% lidocaine was dropped on the eye of the rat until the blinking reflex disappeared. Twenty rats were randomly chosen for keratitis disease establishment. Briefly, an ophthalmic scalpel was used to make an 8 mm^2^ area defect on the cornea. Then, 1 × 10^7^ CFU bacterial was dropped on the eye of rats and remained for 10 min infection. The normal rats were not given the scratch and bacterial infection after the same anesthesia. On the next day, the rats with corneal opacification were identified as successful keratitis modeling. The rats were then treated with different MNs. All the rats in each group were photographed under a microscope and scored according to clinical scoring criteria. On day 3, the cornea of infectious rats was collected in the PBS and the bacterial numbers were measured through agar plate. On day 7, the cornea was collected and embedded in the paraffin, further cut into the 5‐µm thick section for H&E, Masson, and immunohistochemistry staining. The blood of rats was collected for white blood cell (WBC) level analysis. The fresh cornea was collected and fully ground in distilled water. The samples were collected after centrifugation for VEGF‐level analysis via ELISA assay.

### Statistical Analysis

All of the results in this study were presented as mean ± standard deviation (SD), and analyzed by the software GraphPad Prism 8.0. The statistical analysis was evaluated by using a two‐sided Student's *t*‐test or an analysis of variance (ANOVA). The value *p* < 0.05 was recognized as statistically significant.

## Conflict of Interest

The authors declare no conflict of interest.

## Supporting information



Supporting Information

## Data Availability

The data that support the findings of this study are available from the corresponding author upon reasonable request.
