# Supplementary material for: A Transformative Wearable Corneal Microneedle Patch for Efficient Therapy of Ocular Injury and Infection
Source: Adv Sci (Weinh). 2025 Jan 31;12(12):2414548. doi: 10.1002/advs.202414548 (PMC11948004; doi:10.1002/advs.202414548)
Supplement: Supplementary file 1 — Supporting Information [file ADVS-12-2414548-s001.docx]

**Supporting Information**

**A Transformative Wearable Corneal Microneedle Patch for Efficient Therapy of Ocular Injury and Infection**

Xue Jiang^1#^, Shuhua Liu^1#^, Jiayi Chen^1^, Jiapeng Lei^1^, Wenjing Meng^1^, Xueyang Wang^1^, Zhigang Chu^1*^, Wei Li^1,2,3*^

1. Department of Burns, Tongren Hospital of Wuhan University (Wuhan Third Hospital), School of Pharmaceutical Sciences, Wuhan University, Wuhan 430071, China.

2. TaiKang Center for Life and Medical Sciences, Wuhan University, Wuhan, China.

3. Hubei Provincial Key Laboratory of Developmentally Originated Disease, Wuhan 430071, China

# These authors contribute equally to this work.

* Corresponding authors: E-mail address: weili.mn@whu.edu.cn (Wei. Li); chuzhg@hotmail.com (Zhigang. Chu).


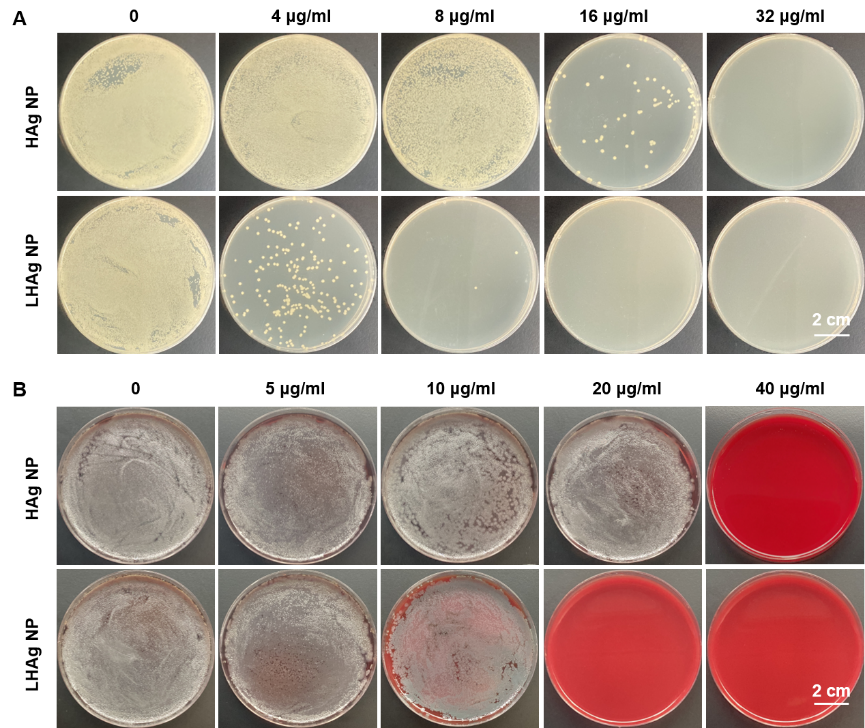


**Figure S1**. The agar plate of *S. aureus* (**A**) and *Streptococcus* (**B**) incubated with different concentration of HAg NP or LHAg NP.


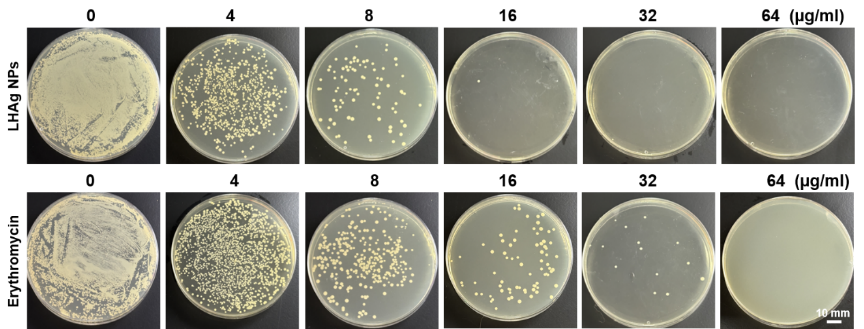


**Figure S2.** The antibacterial experiment of LHAg NPs and erythromycin on drug-resistant *S. aureus*.


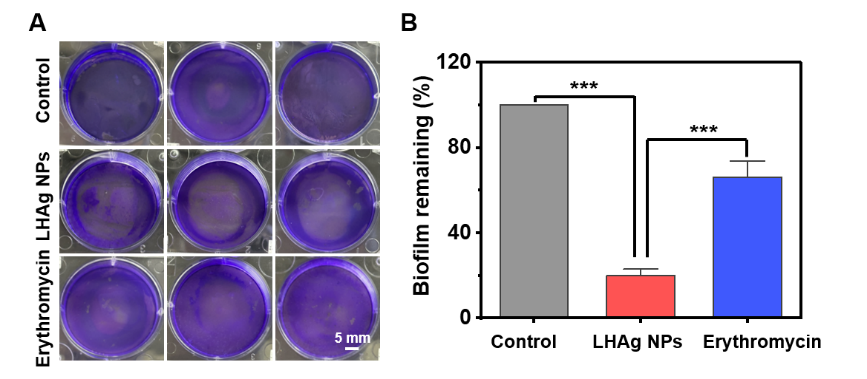


**Figure S3.** (A) Crystal violet staining of biofilm. (B) Quantification of biofilm remaining after different treatments (n = 3). All data are represented as mean ± SD. (****P* < 0.001).


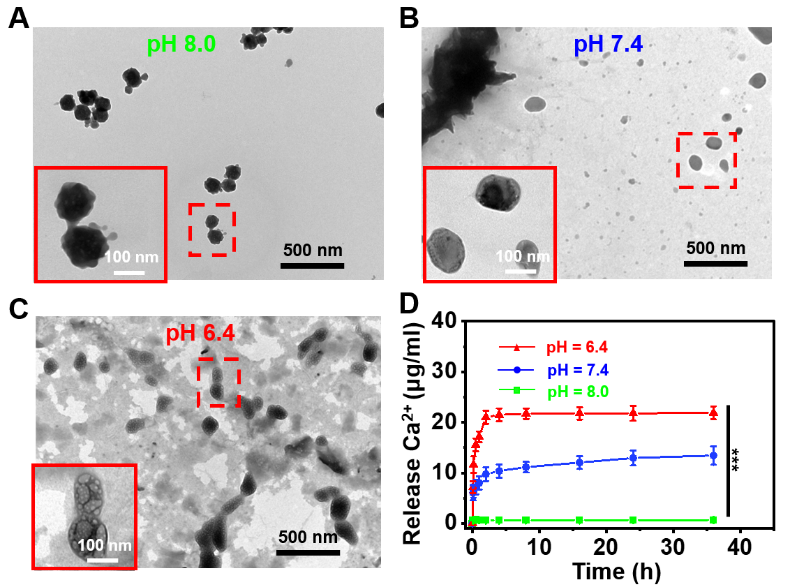


**Figure S4**. (**A-C**) TEM images of CaCO_3_ NPs after incubation in different pH medium for 10 min. (**D**) The Ca^2+^ release from CaCO_3_ NPs at different pH condition (n = 3). All data are represented as mean ± SD. (****P* < 0.001).


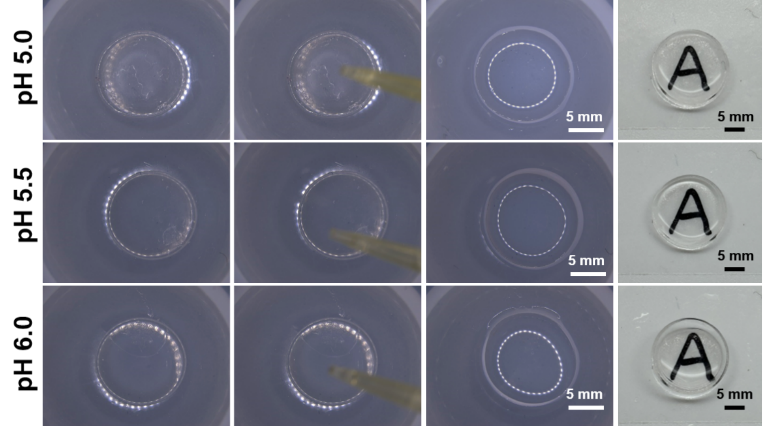


**Figure S5**. The gelation process of hydrogel at pH 5.0, 5.5 and 6.0, respectively.


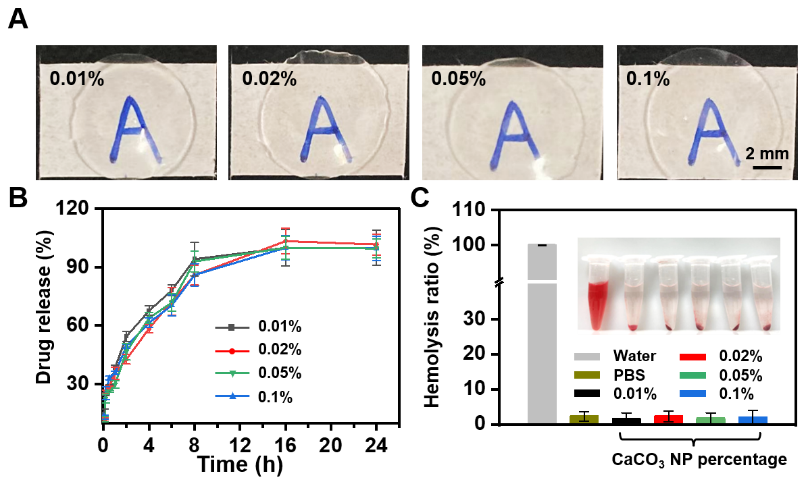


**Figure S6.** (**A**) SSA/Ca film containing different concentration of CaCO_3_ NPs (0.01%, 0.02%, 0.05%, or 0.1%). (**B**) Drug release from SSA/Ca hydrogel containing different concentration of CaCO_3_ NPs (n = 3). (**C**) Hemolysis ratio of SSA/Ca hydrogel containing different concentration of CaCO_3_ NPs (n = 3). All data are represented as mean ± SD. (****P* < 0.001).


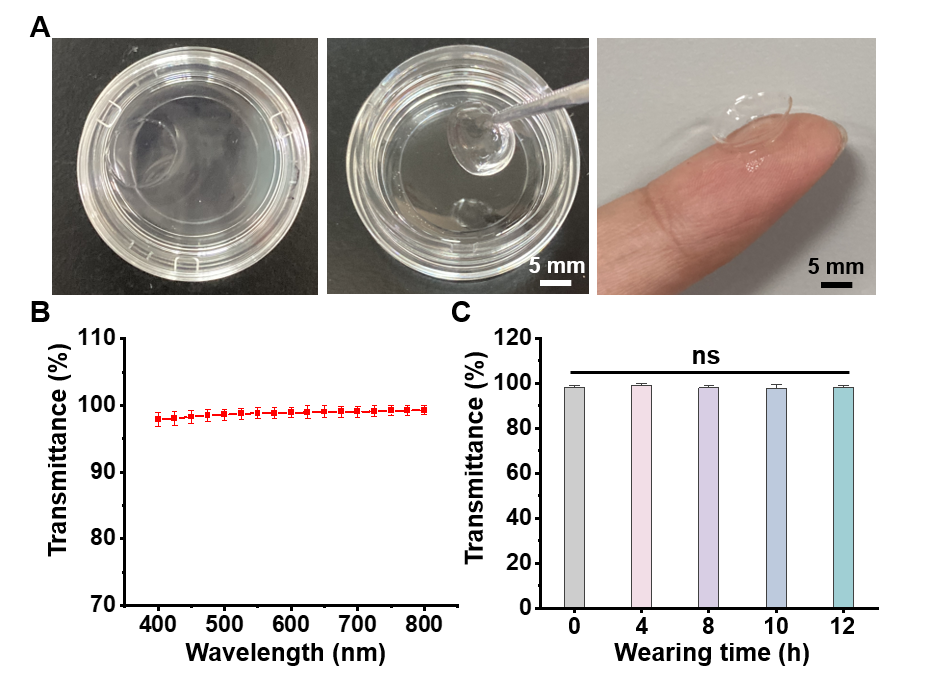


**Figure S7.** (**A**) The immersion of the hydrogel in tear simulated buffer for 72 h. (**B**) The quantification of transmittance of the hydrogel after immersion in tear simulated buffer for 72 h (n = 3). (**C**) The transmittance changes of the hydrogel after being worn on rat corneas for different time periods (n = 3). All data are represented as mean ± SD. The n indicates no significance.


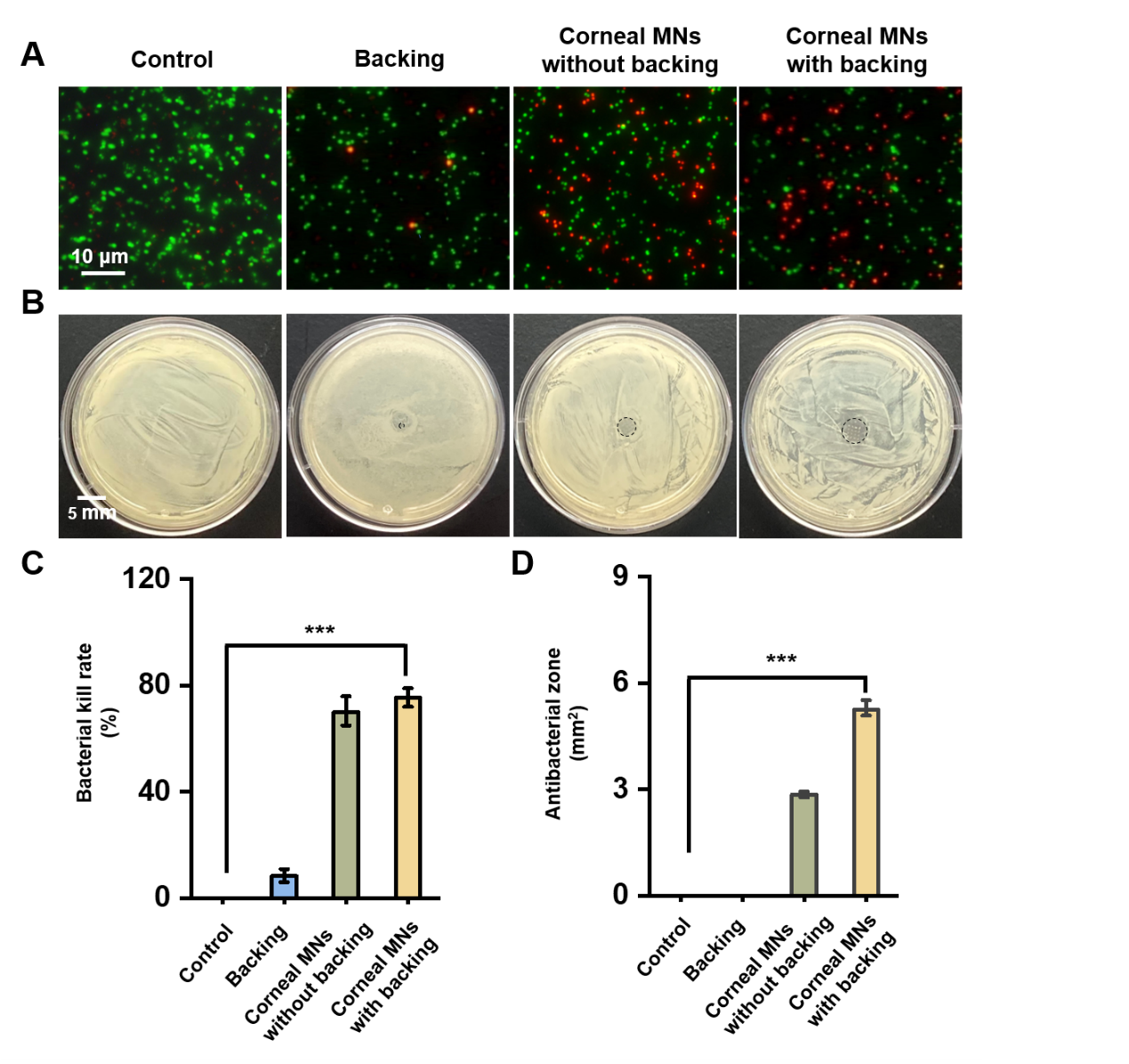


**Figure S8**. (**A**) The SYTO9/PI staining images of *S. aureus* after receiving different treatments. (**B**) The inhibition zone of *S. aureus* after receiving different treatments. (**C**) The bacterial kill rate of *S. aureus* after receiving different treatments. (n = 3). (**D**) The analysis of bacteriostatic zone area (n = 3). All data are represented as mean ± SD. (****P* < 0.001).


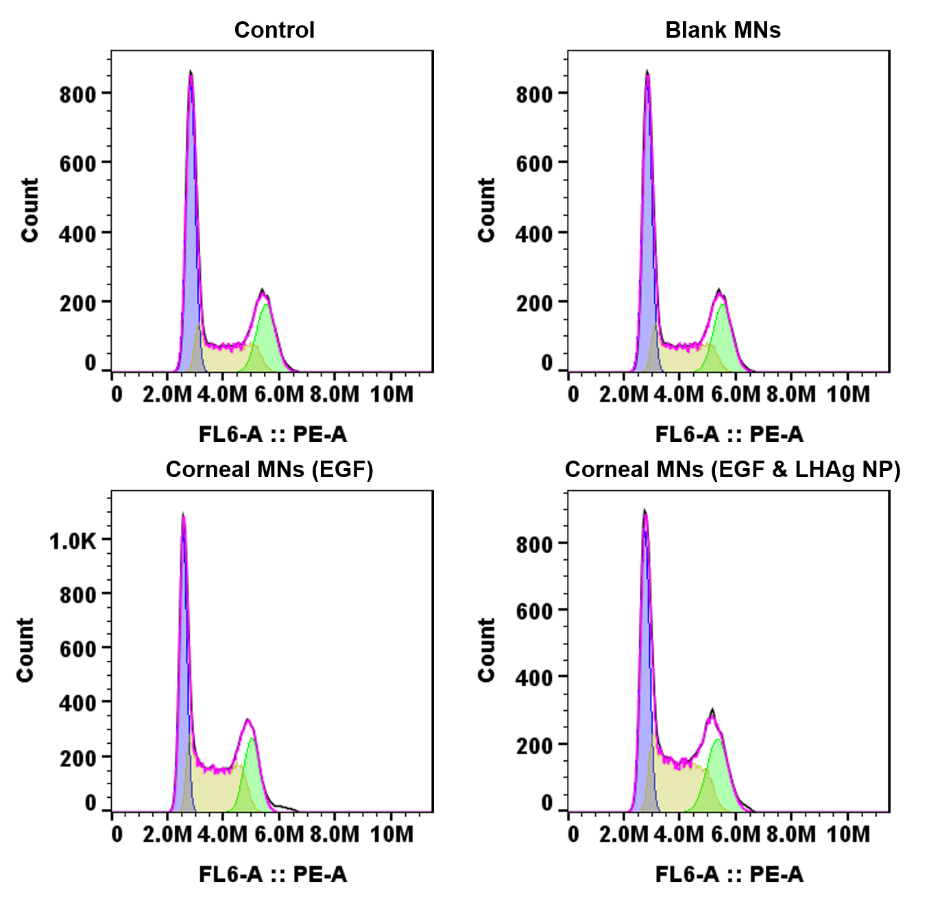


**Figure S9**. The fitted cell cycle of HCEC cells after incubation with different MNs.


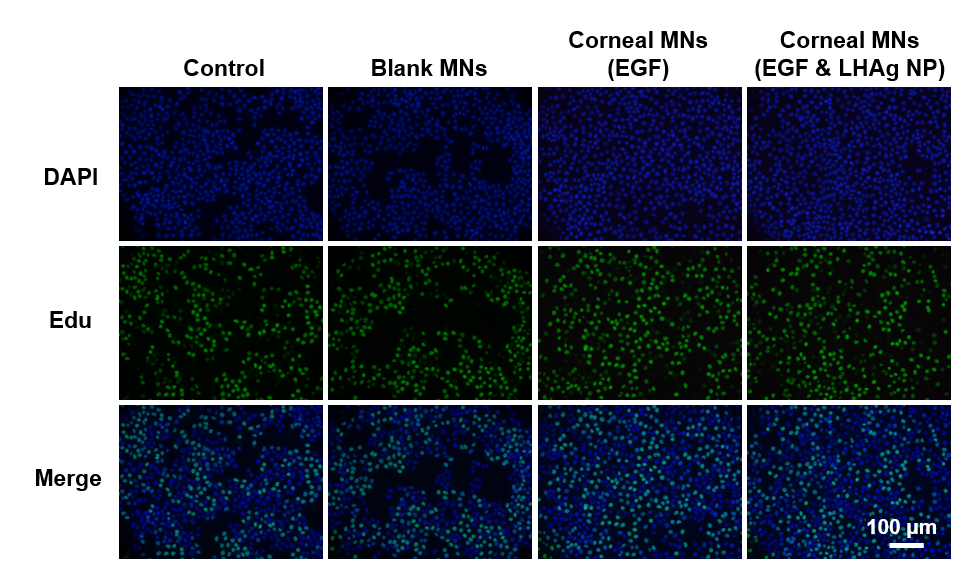


**Figure S10**. The staining images of HCEC cells after incubation with different MNs.


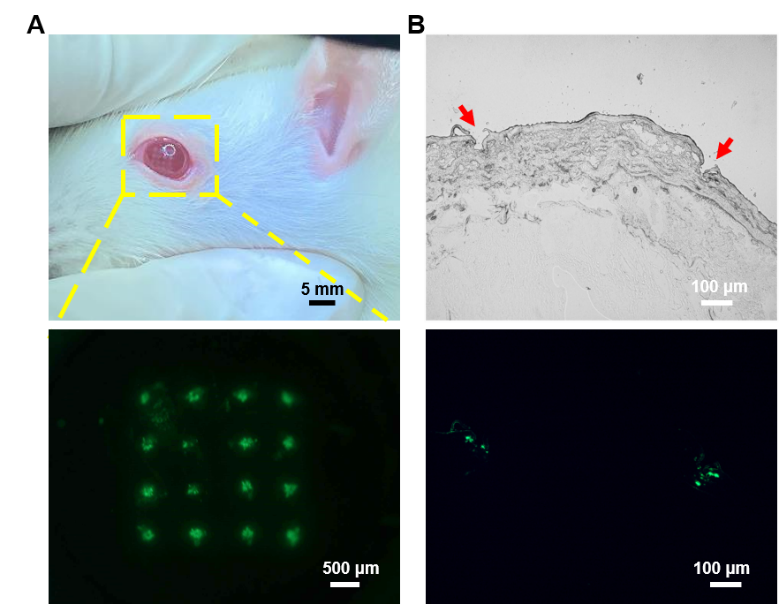


**Figure S11.** (**A**) The insertion of MNs in rat eyeball in vivo. (**B**) Histological section image of rat cornea after corneal insertion of the MNs. The red arrowheads indicate the penetration holes of the MNs.


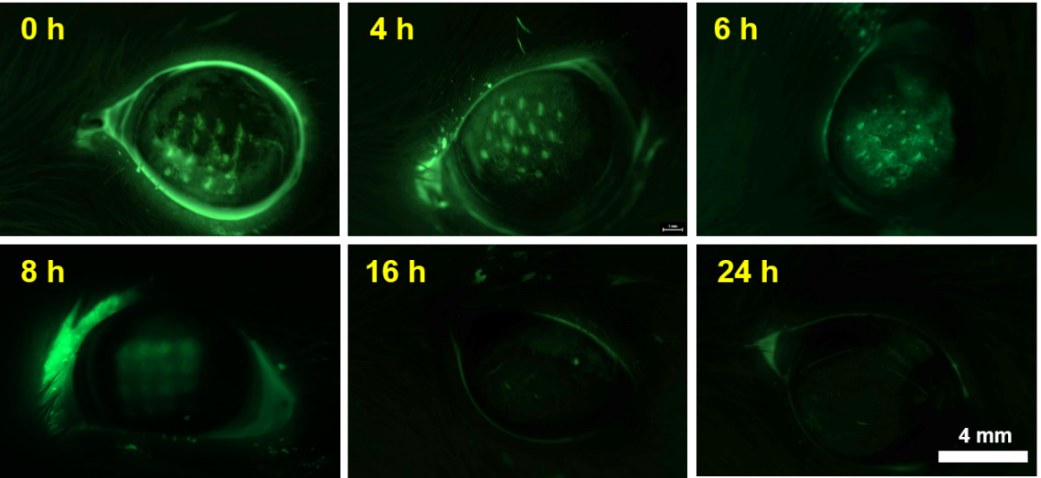


**Figure S12.** Micropores closure after application of the transformative MN patch in rat cornea in vivo.


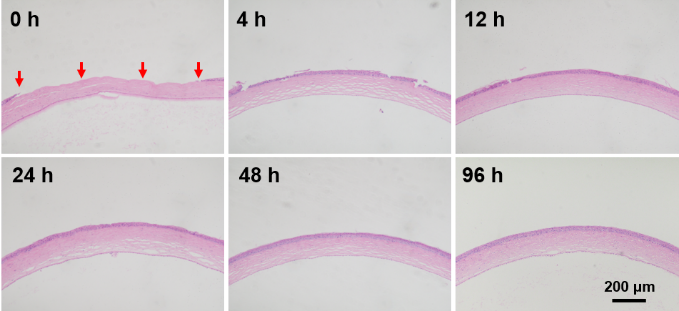


**Figure S13**. H&E staining of rat corneas after treatment with the MN patch for corneal wound healing. Red arrowheads indicate the location of the scratch on the cornea after the injury model was created.


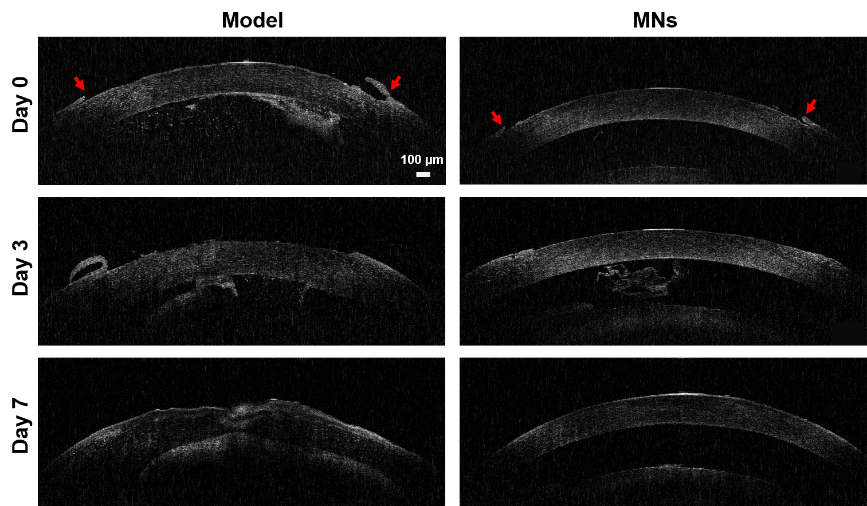


**Figure S14.** OCT images of rat corneas from day 0 to day 7 after receiving the treatment of the MN patch. The model group was used as a control that did not receive any treatment after injury. Red arrowheads indicate the location of the scratch on the cornea after the injury model was created.


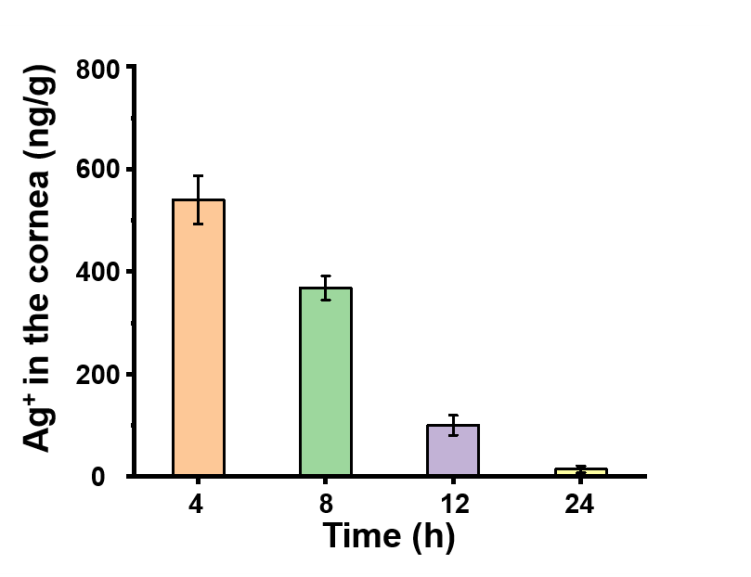


**Figure S15.** The release of Ag ions from LHAg NPs in the cornea (n = 5). All data are represented as mean ± SD.


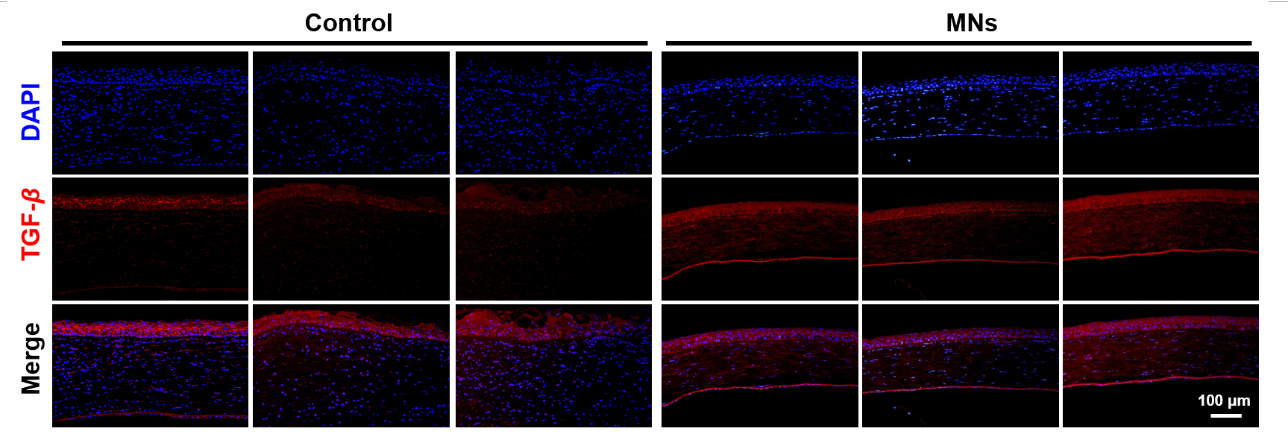


**Figure S16.** Immunofluorescence staining of TGF-*β* 5 days after administration of the MN patch in rat corneas in vivo.


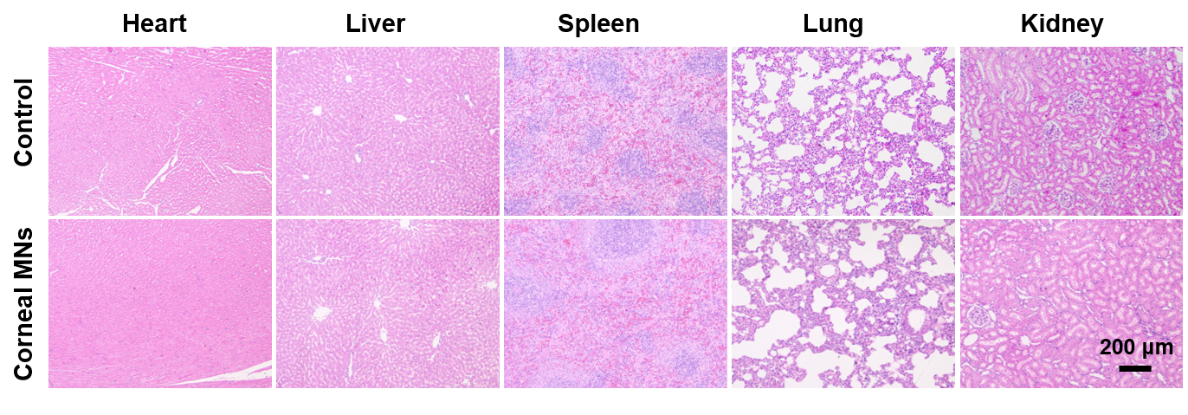


**Figure S17**. Biosafety of the corneal MN patch on heart, liver, spleen, lung, kidney.
